# Bell’s palsy with preeclampsia in pregnancy

**DOI:** 10.1002/ccr3.5918

**Published:** 2022-05-23

**Authors:** Yeshey Dorjey

**Affiliations:** ^1^ Phuentsholing Hospital Chukha Bhutan

**Keywords:** antiviral drug, bell's palsy, corticosteroid, preeclampsia

## Abstract

Bell's palsy in pregnancy is not frequently seen. The association of preeclampsia with Bell's palsy is reported in the research, however, the exact link between Bell's palsy and preeclampsia is unknown. The treatment of Bell's palsy during the pregnancy is difficult and controversial. We report two cases of Bell's palsy with preeclampsia diagnosed during the third trimester of the pregnancy. The first case was diagnosed with Bell's palsy with severe preeclampsia with placental abruption with fetal distress; the pregnancy was terminated by cesarean section. Another case was diagnosed with Bell's palsy with mild preeclampsia. She had a spontaneous vaginal delivery at term. Both the cases achieved complete recovery from Bell's palsy after treatment with corticosteroid and antiviral drugs. Every obstetrician should be able to recognize Bell's palsy in pregnancy and initiate early treatment with corticosteroid and mount surveillance for preeclampsia.

## Graphical abstract

There is likely an association of Bell's palsy with preeclampsia during pregnancy. It is imperative to initiate screening for preeclampsia in pregnant women with Bell's palsy to detect and treat it early to improve the maternal and perinatal outcomes.

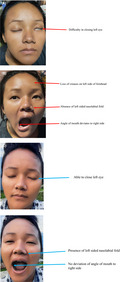

## 1 INTRODUCTION

Bell's palsy is a form of temporary facial paralysis or weakness on one side of the face. It is also known as idiopathic facial palsy. The seventh cranial nerve (facial nerve) supplies muscles of the face, eye, tear glands, salivary glands, and muscles of the small bones in the middle ear, and it transmits taste sensation from the tongue. The dysfunction of this seventh cranial nerve (facial nerve) results in Bell's palsy.^1^ Idiopathic facial palsy or Bell's palsy is the most common cause of facial paralysis.^2^ It has been postulated the likely association of viral infections or reactivation of herpes simplex virus, varicella‐zoster virus, cytomegalovirus, Epstein‐Barr virus, and human herpes virus‐6 with Bell's palsy although it is still considered as a diagnosis of exclusion.^3^, ^4^ The viral infection causes inflammation and edema in the bony fallopian canal or the facial nerve canal and results in peripheral facial palsy.^5^ The facial nerve canal is the longest and narrow bony canal in the body through which the facial nerve traverses the petrous temporal bone from the internal acoustic meatus to the stylomastoid foramen.^6^

A high extracellular fluid content as part of physiological changes, hypercoagulable states, hormonal changes, and relative immunosuppression in pregnancy, the edema in preeclampsia, viral inflammation, and edema are thought to be predisposing factors for the development of Bell's palsy.^5^, ^7^

The incidence of Bell's palsy is around 40 persons per 100,000 women in pregnancy which is high as compared to non‐pregnant women and it is seen more frequently during the third trimester and the immediate postpartum period.^7^, ^8^

In general, Bell's palsy is a unilateral facial paralysis affecting only one side of the face, and in rare instances, it can affect both sides.^9^, ^10^ The symptoms appear within a 2–3 days period and generally, the majority improves with treatment completely; however, few permanent neurological paralyzes of the facial nerve were reported.^11^

The treatment of Bell's palsy in pregnancy remains controversial. The American Academy of Neurology and the other literature recommend treatment with corticosteroids alone.^12^, ^13^ However, combined therapy of corticosteroid with the antiviral drug is being used frequently in treating a severe form of facial paralysis.

This write‐up is to describe the cases of Bell's palsy with preeclampsia in pregnancy diagnosed during the third trimester and treated with combined therapy.

## 2 CASE PRESENTATIONS

### 2.1 Case‐1

**FIGURE 1:**
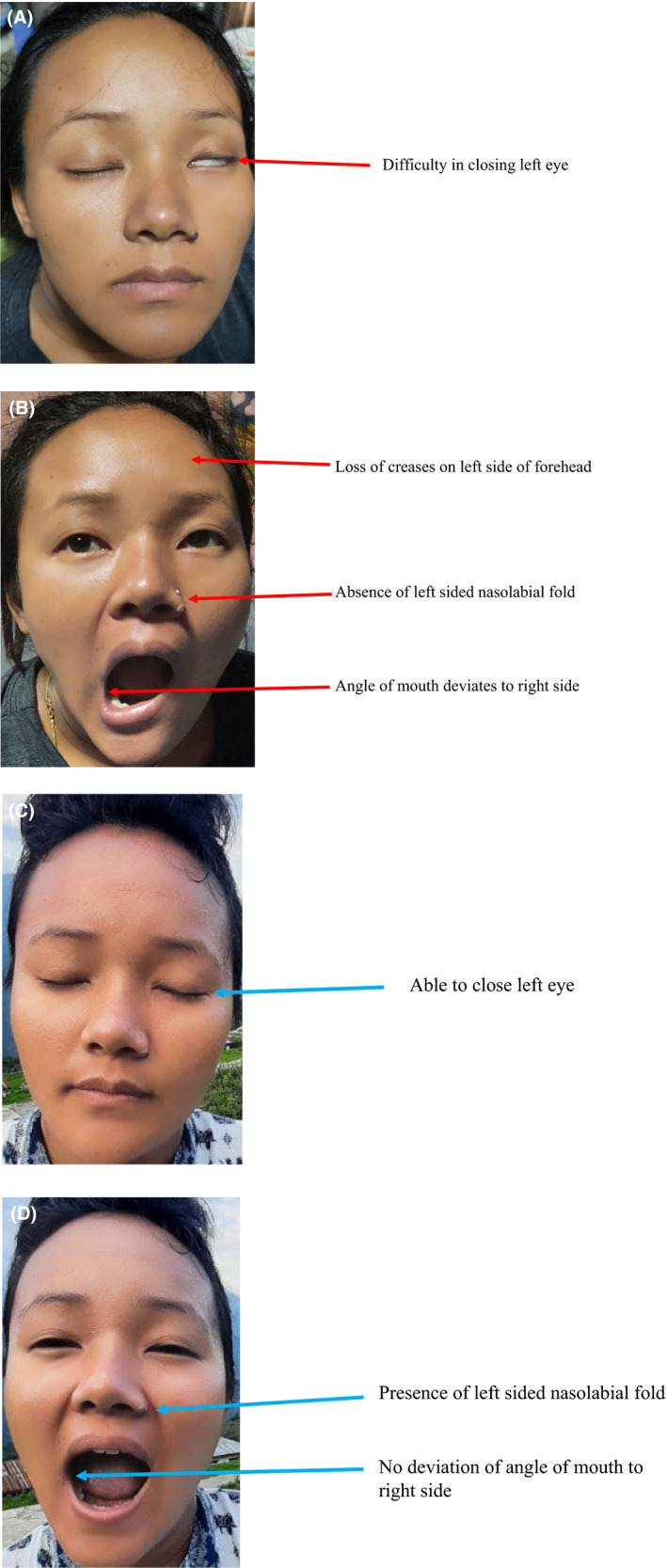
Image of the patient. Images (A,B) show features of Bell's palsy present before the treatment (red arrows); Image (C,D) show complete recovery of the Bell's palsy features after the treatment (blue arrows)

A 29‐year‐old, G2P1 at 31^+4^ weeks gestation was admitted to the maternity ward on August 19, 2020 with complaints of sudden onset of left‐sided facial weakness, difficulty in closing the eye on the left side for 2 days, and mild abdominal pain for a 1‐day duration. She does not gave a history of trauma, ear pain, ear discharge, fever, or loss of taste sensation. She was complaining of a mild headache, however, no blurring of vision or chest pain. This was a planned pregnancy and her antenatal care (ANC) follow‐up was uneventful until the development of the above symptoms. Her neurological examination of the face revealed a loss of creases on the left side of the forehead, inability to close her left eye, wide palpebral fissure, weakness of muscle on the left side of the face, deviation of the angle of mouth on the right side, and loss of left nasolabial fold (Figure 1A,B). Corneal reflexes showed a delayed eyelid closure on the left side. There was slight slurring of speech. On the external auditory canal and the otoscopic examination, there was no rash, vesicle, erythema, or discharge. There was no swelling or mass in the parotid gland. The neurological examination of the upper and lower limbs was unremarkable.

Her pulse rate was 98 beats/min, BP 160/112 mm Hg, respiratory rate 18 breaths/min, SpO_2_ 96%, and her urine output was adequate. On obstetrical examination, symphysis fundal height was 32 cm and it was corresponding to the period of amenorrhea, uterus was soft with mild tenderness noted, fetal parts are felt and the fetal heart sound was audible. On per vaginal examination, Bishop's score was unfavorable and there was no per vaginal bleeding. The bedside urine dipstick test for protein showed 3^+^ for proteinuria. The hematological tests showed creatinine 1.0 mg/dl, platelet count 125,000/μl, AST 34 UI/L, ALT 40 UI/L, and viral markers are all negative. The cardiotocography was performed and fetal heart rate tracing was reassuring. From the above findings, the patient was diagnosed with Bell's palsy with severe preeclampsia.

Blood pressure was controlled with Inj. Labetalol; for fetal lung maturity corticosteroid Inj. Dexamethasone 6 mg IM 12 hourly four doses were given and closed fetal and maternal assessments were done.

**FIGURE 2:**
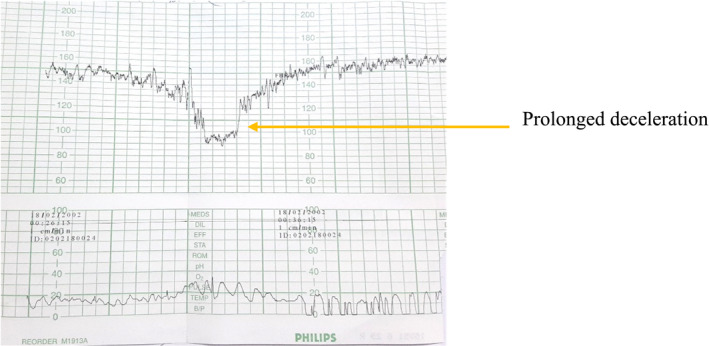
CTG showing prolonged deceleration

On August 21, 2020 patient was complaining of severe headache, abdominal pain, and reduced fetal movement. On clinical assessment, she was mildly anemic, SpO_2_ 96%, RR 20 breath/min, PR 100 beats/min, BP 180/116 mm Hg, and urine output was adequate. On obstetrical examination, there was a tender, rigid, contracted uterus, fetal parts not felt, and fetal heart sound was feebly audible; the cardiotocography showed prolonged deceleration present (Figure 2).

On vaginal examination, the cervix was unfavorable and per vaginal bleeding was not found. Ultrasonography was performed and placental abruption could not be excluded. Prophylactic Inj. MgSO_4_ 4g IV over 20 min (loading dose) and MgSO_4_ IV 1 g/h (maintenance dose) were administered for dual purposes; prevention of eclampsia and fetal neuroprotection. After the stabilization of the mother, the pregnancy was terminated by emergency cesarean section under spinal anesthesia on August 21, 2020. Per‐operatively there was a massive retroplacental blood clot with an umbilical cord double around the neck of the baby found. A live male baby weighing 1600 g was delivered with an Apgar score of 9 in 1 min and 10 in 5 min. After the cesarean section, the patient was treated with Tab. Prednisolone 40 mg daily for 7 days, then tapered doses over the next 12 days. Tab. Acyclovir 400 mg 5 times daily for 10 days was given with prednisolone along with daily physiotherapy for Bell's palsy. Her blood pressure was within normal range after the delivery. Mother was discharged on postoperative day 8. The baby was suspected to have retinopathy of prematurity and was referred to Jige Dorji Wangchuk National Referral Hospital, Thimphu for further evaluation and management. Follow‐up after 10 months showed the patient is still feeling slight weakness on the left side of the face, however, on clinical examination, the finding was unremarkable (Figure 1C,D).

### 2.2 Case‐2

A 29‐year primigravida at 32^+5^ weeks gestation was admitted to the maternity ward on August 24, 2020 with complaints of sudden onset of right‐sided facial weakness and difficulty in closing the eye on the right side for a 1‐day duration. She has no history of trauma, ear pain, ear discharge, fever, or loss of taste sensation. She did not have similar facial paralysis in the past. Her ANC follow‐up was uneventful and she was normotensive until the development of the above symptoms. Her neurological examination showed a loss of wrinkles on the right forehead, inability to close her right eye, weakness on the right side of the face, angle of mouth deviating to the left side, and loss of right nasolabial fold. Corneal reflex was slow on the right side. There was no slurring of speech. On examination, there were no rashes, vesicles, erythema, or discharge from the ear. The parotid gland was normal and the neurological examination of the upper and lower limbs was unremarkable. Her BP was 150/100 mm Hg, PR 90 beats/min, and RR 14 breaths/min. On obstetrical examination no significant finding. A pelvic examination was not performed. The bedside urine dipstick test showed 1^+^ for proteinuria. The fetal heart rate tracing with cardiotocography was reassuring. Her renal and liver function tests were within the normal range for the pregnancy. With the above findings, she was diagnosed with Bell's palsy with mild preeclampsia. The patient was treated with Tab. Prednisolone 60 mg PO daily for 7 days, then tapered over the next 12 days. Tab. Acyclovir 400 mg 5 times daily for 10 days was given in combination with the prednisolone in addition to daily physiotherapy. Her BP control was achieved with Tab. Nifedipine 20 mg given 12 hourly. She was discharged home with the above oral medications and had a closed follow‐up at the Maternal and Child Health care (MCH) unit. Her BP was under control and there was an obvious clinical improvement in Bell's palsy during the follow‐up visits. She had a spontaneous vaginal delivery at term on October 22, 2020 and delivered a healthy female baby weighing 3900 g. The patient had achieved full recovery of Bell's palsy after 6 weeks of the treatment.

## 3 DISCUSSION

Among the cranial nerves, the facial nerve is a special one as it has to travel a long distance through a tiny narrow bony tunnel with little room for swelling.^6^ As the facial nerve has to travel through a narrow bony canal, the slightest inflammation from any cause easily produces compressive neuropathy that manifests as facial nerve dysfunction.^4^

In the present cases, both the pregnant mothers developed Bell's palsy in their third trimesters of the pregnancy. In the literature, Bell's palsy was reported in the third trimesters and early postpartum period.^14^, ^15^ Bell's palsy in the first trimesters of the pregnancy is an extremely rare occurrence.^9^ Both the pregnant mothers reported here had Bell's palsy with preeclampsia. Previously it was postulated the possible association of Bell's palsy with preeclampsia during the pregnancy. The extracellular fluid retention and the edema of preeclampsia are the possible explanation for the association of Bell's palsy with preeclampsia.^16^

The treatment of Bell's palsy during the pregnancy is controversial and challenging. There is no standard recommendations or guideline on the treatment of Bell's palsy during the pregnancy. The concern of adverse effects of drug use during pregnancy on the fetus makes the treatment decision more difficult. The multicentric clinical trials have shown corticosteroid, prednisolone alone showed superiority over the combined therapy of prednisolone with antiviral, acyclovir, or use of acyclovir alone.^17^ However, an additional synergistic effect was observed when acyclovir was used in combination with prednisolone for the treatment of severe Bell's palsy.^13^ Bell's palsy in pregnancy recovers fully with the treatment. A better recovery of Bell's palsy is seen when treated with a high dose (≥450 g) as compared to a low dose (<450 g) of prednisolone.^13^ Both the patients in the present cases were administered a high dose of prednisolone (≥450 g) and both of them had a full recovery after the treatment. The recovery is faster with the initiation of steroid treatment within 24–48 h of the onset of symptoms. Bell's palsy in pregnancy does not increase adverse perinatal outcomes.

## 4 CONCLUSIONS

Every obstetrician should be aware of the unexpected neurological complications and be able to recognize Bell's palsy and initiate treatment with steroids and perform surveillance for preeclampsia.

## AUTHOR CONTRIBUTIONS

YD is involved in the conception, consent, data collection, manuscript writing, and submission for publication.

## CONFLICT OF INTEREST

The author does not have a conflict of interest to declare.

## CONSENT

The informed written consent has been obtained from the patient to collect the case history, collect the data, take the pictures, and write a case report for publication in the medical journals.

## Data Availability

The data that support this writing is available from the corresponding author upon reasonable request.
